# Antibody Testing for Neurological Autoimmune Disorders: Evaluation of Best Practices at a Tertiary Referral Center

**DOI:** 10.3389/fneur.2021.690415

**Published:** 2021-06-30

**Authors:** Sarah E. Fredrich, Steven Vernino, Kyle M. Blackburn

**Affiliations:** Department of Neurology, University of Texas Southwestern Medical Center, Dallas, TX, United States

**Keywords:** autoimmune neurology, stewardship, antibody panels, evaluation of paraneoplastic disorders, evaluation of encephalitis, repeat testing, utilization, practice patterns

## Abstract

**Background:** Autoimmune neurology is a rapidly evolving field of study, where best practices for neurological antibody testing have yet to be determined. The growing number of options for antibody panel testing can create confusion amongst ordering clinicians and lead to ordering several concurrent panels (i.e., overlapping evaluations) or repeat panel evaluations. This study determined the frequency of these evaluations for autoimmune and paraneoplastic disorders and investigated how these practices informed clinical decision making and management.

**Methods:** This was a retrospective observational study of adult patients presenting to University of Texas Southwestern (UTSW) in 2017 with requests for antibody panels for autoimmune encephalitis and paraneoplastic disorders. Individuals with more than one panel requested were defined as either an overlapping evaluation (more than one panel requested within 14 days) or repeat evaluation (more than one panel requested 14 or more days apart). For those individuals with repeat panel testing, the proportion of panels with a change in antibody status or subsequent changes in clinical diagnosis and decision making were recorded.

**Results:** There was a total of 813 panels sent on 626 individuals. Twenty percent (126 individuals) had more than one panel requested. Only 10% of individuals had a matched serum and CSF evaluation. Forty-seven overlapping evaluations were performed in 46 (7.3%) of the individuals studied. Fifty-four (8.6%) individuals underwent 70 repeat evaluations encompassing 79 panels (9.7% of total panels ordered). Ten repeat evaluations showed a change in antibody status, of which only two were clinically significant. There was a single case where clinical management was affected by repeat autoantibody evaluation.

**Conclusions:** Ordering practices for suspected autoimmune encephalitis and paraneoplastic disorders are suboptimal with frequent overlapping antibody panel evaluations and non-paired serum/CSF samples at our center. Repeat autoantibody testing is a commonplace practice yet yielded novel information in only a minority of cases. These new results were, as a rule, clinically irrelevant and changed clinical decision making in <1% of cases. There is limited utility in these practice patterns. Future efforts should be directed at the development and standardization of neurological autoimmune and paraneoplastic autoantibody testing practice standards.

## Introduction

Autoimmune neurology is a rapidly evolving field of study, largely fueled by the discovery of autoantibodies targeting neuronal and glial proteins. Antibody-mediated neurological disorders typically present with severe, progressive neurological symptoms, and timely treatment with immunotherapy can result in dramatic improvement and favorable long-term outcomes (1). Antibody testing for autoimmune and paraneoplastic neurological disorders is available through several commercial laboratories in the United States. Best practices for neurological antibody testing have not been defined, but it is generally advised that both serum and cerebrospinal fluid (CSF) samples be submitted for testing, as certain antibodies are more sensitive in CSF (2). In addition, different neuronal autoantibodies can present with overlapping clinical features, antibody “panels” are commonly ordered for suspected autoimmune neurological disorders. The growing options for antibody testing can create confusion amongst ordering clinicians, leading them to order multiple panels during a single encounter to ensure a comprehensive evaluation. Additionally, antibody testing is occasionally repeated in the same patient during a future patient encounter. Whether these practices increase detection of antibody-mediated neurological disorders has not been systematically examined. In this study, we determined the frequency of overlapping and repeat antibody testing for autoimmune and paraneoplastic disorders in patients presenting to the University of Texas Southwestern Medical Center (UTSW) and investigated how these practices informed clinical decision making and management.

## Materials and Methods

**Figure 1:**
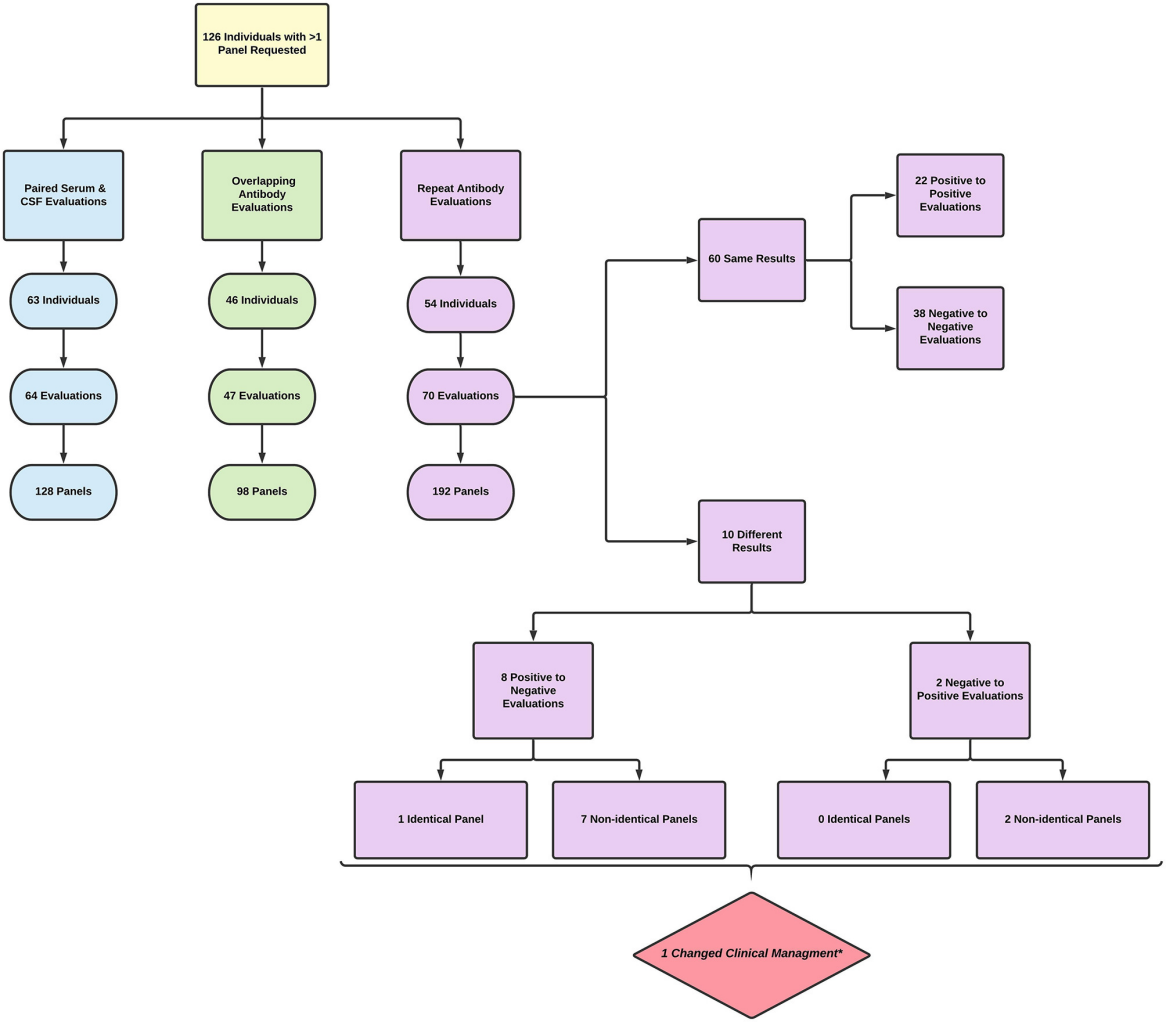
Flowchart of results for individuals with >1 antibody panel requested.

The study was approved by the UTSW institutional review board. This was a retrospective observational study of adult patients (>18 years old) presenting to UTSW in 2017 with requests for autoimmune encephalitis, epilepsy, dementia, and paraneoplastic disorders antibody panels sent to Mayo Medical Laboratories^1^. These patients were cross matched with a list of requests for antibody testing submitted to the UTSW clinical laboratory from 2011 to 2020. Patients from 2017 with more than one antibody test requested during this period were included in the final analysis (see Figure 1).

Instances where more than one panel was requested were placed in one or more of the following categories: paired serum and CSF evaluation, overlapping evaluation, and/or repeat evaluation. A paired serum and CSF evaluation was defined as a request for serum and CSF panels obtained within 14 days of each other. An overlapping evaluation was defined as multiple requests for antibody panels obtained <14 days apart, excluding paired serum and CSF evaluations. A repeat evaluation was defined as more than one antibody panel obtained ≥14 days apart. Accidental reordering was defined as multiple requests for identical antibody panels <14 days apart. The 14-day time period was chosen to demarcate overlapping and repeat testing as, in our experience, results from initial testing tend to return in this timeline.

For those individuals with repeat autoantibody evaluations, the results of the panel testing were recorded. The number of results with a change in antibody status (e.g., going from antibody positive to negative) was tabulated. A single evaluation was considered positive if any of the individual panels returned with a positive result. If a single individual had more than one repeat evaluation, the additional repeat evaluations were compared to the most recent evaluation for the purpose of determining change in antibody status. If an antibody status change was found, this was sorted into one of four categories based on comparison of identical or non-identical panels and positive to negative or negative to positive panel transition (see Figure 1). The time between repeat evaluations was determined by the date of the last panel in the first evaluation to the date of the first panel in the second evaluation.

For individuals who had one or more repeat evaluations with a change in autoantibody status, medical records were reviewed for changes in clinical diagnosis and medical decision making based on repeat panel results. Changes in medical decision making were defined as one or more of the following: starting, stopping, or changing immunotherapies, starting or stopping anti-seizure medications (ASM) in a patient who presented with seizure, ordering a malignancy screening consisting of positron emission tomography (PET) scan and/or computed tomography (CT) scan of the chest, abdomen, and pelvis, within 30 days of panel results. Clinical diagnoses were abstracted from neurology consultation notes and billing codes.

## Results

**Figure 2:**
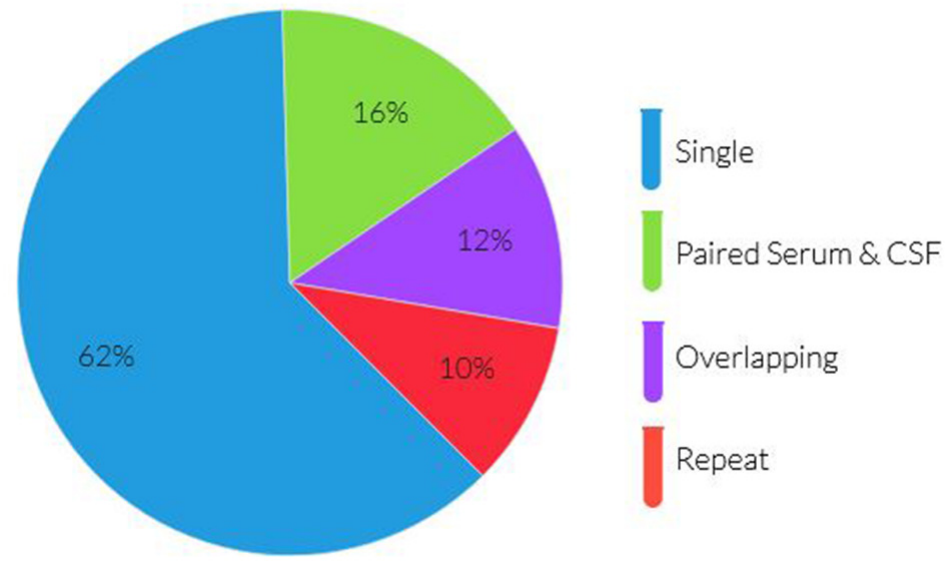
Proportion of panels by evaluation type.

**Figure 3:**
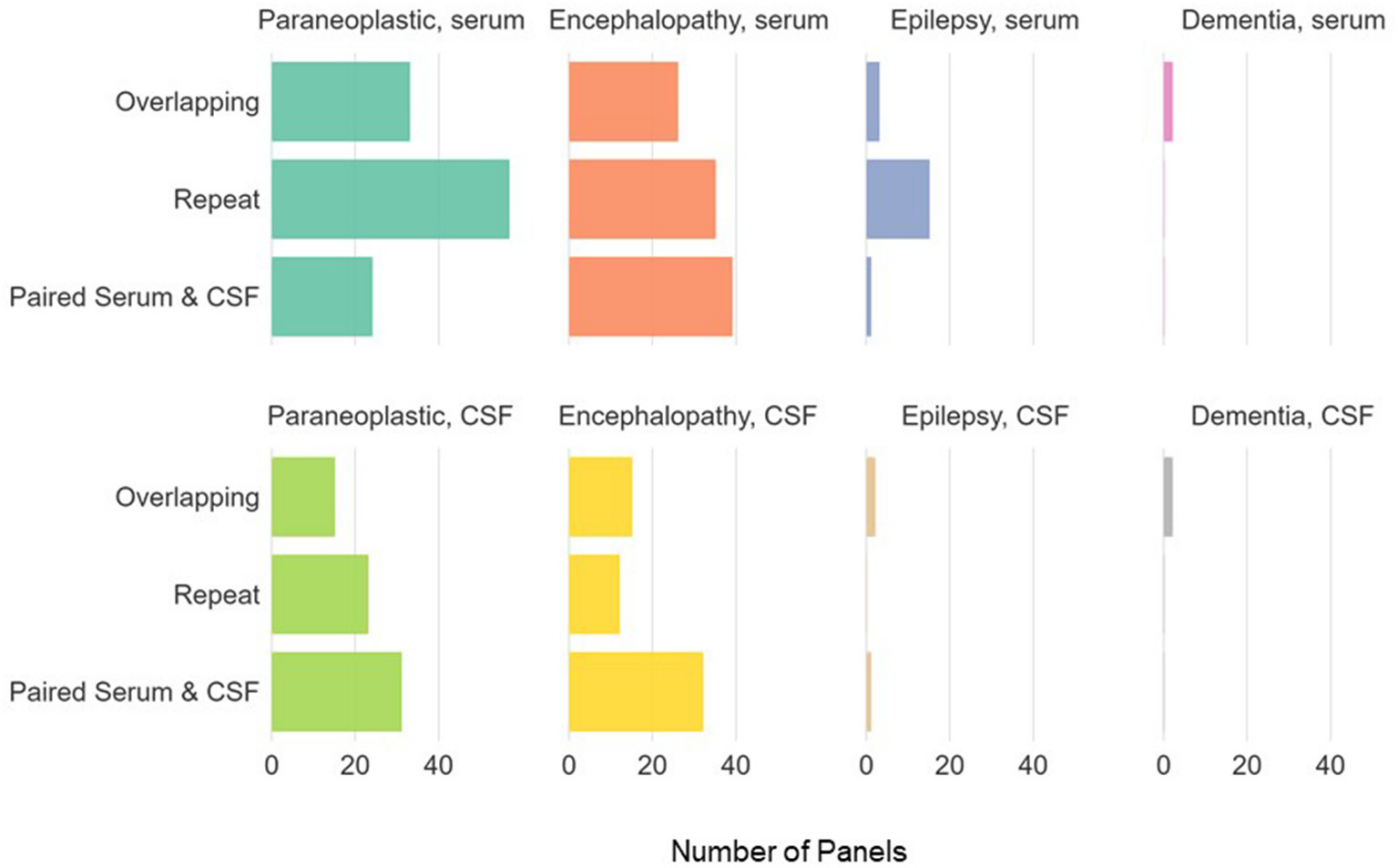
Panel types ordered for individuals with >1 antibody panel requested. Paraneoplastic panels include test codes PAVAL, PAC1. Encephalopathy panels include test codes ENS1, ENS2, ENC1, ENCEC, ENCES. Epilepsy panels include test codes EPC1, EPS1, EPS2, EPIES, EPIEC. Dementia panels include test codes DEMEC, DEMES.

There was a total of 768 antibody panels submitted on 626 individuals in 2017. The average age of this group was 57.2 years (ranging from 18 to 93 years old) with slight female predominance (53% of individual). Ultimately 24 cases were due to autoimmune or paraneoplastic disorders. One hundred and twenty six individuals (20.1%) had more than one panel requested (see Figure 1). The proportion of panels and evaluation types can be seen in Figures 2, 3. There were an additional 45 panels ordered as repeat antibody evaluations from 2011 to 2020 on these individuals. In total, 290 panels (35.7% of all panels studied) were requested as part of an overlapping or repeat panel evaluations (see Table 1). Six patients had both overlapping and repeat evaluations performed.

**Table 1:** Number of individuals, evaluations, and panels performed by evaluation type.

**Evaluation type**	**Number of individuals (%)**	**Number of evaluations (%)**	**Number of panels (%)**
Single panel	500 (79.9%)	500 (67.8%)	500 (61.5%)
Paired serum & CSF	63 (10.1%)	64 (8.7%)	128 (15.7%)
Overlapping	46 (7.3%)	47 (6.4%)	98 (12.1%)
Repeat	54 (8.6%)	70 (9.5%)	79 (9.7%)
Identical panels	48 (7.7%)	57 (7.7%)	58 (7.1%)

### Paired Serum and CSF Evaluations

There were 64 paired serum and CSF evaluations (128 panels, 15.7%). Only 10.1% of individuals had a paired serum and CSF evaluation. One individual had two paired serum and CSF evaluations performed.

### Overlapping Evaluations

Forty-seven overlapping evaluations were performed in 46 (7.3%) of the individuals studied. Overlapping evaluations included 98 panels (12.1% of total tests requested). Of these, 17 (36%) overlapping evaluations were requested on identical panels. While the majority of overlapping evaluations consisted of two panels per evaluation, four individuals had overlapping evaluations with three panels ordered. One individual additionally had two overlapping evaluations.

### Repeat Evaluations

Fifty-four (8.6%) individuals underwent 70 repeat evaluations encompassing 79 repeat panels (9.7% of total panels ordered). The average time between repeat evaluations was 350 days (median 200 days, range 140–1,941 days). Identical panels were requested as a part of 41 (58.6%) repeat evaluations. Eight individuals underwent more than one repeat evaluation, with two individuals having three repeat evaluations. Eighty-six percent of repeat evaluations did not show a change in autoantibody status, with 97% of initial negative evaluations remaining negative.

Ten repeat evaluations had a change in antibody status (Figure 1). Eight repeat evaluations (11.4%) changed from positive to negative. One evaluation compared identical panels and seven compared non-identical panels. Two evaluations (2.8%) changed from negative to positive; in both instances, a different panel was ordered in the repeat evaluation (Table 2).

**Table 2:** Repeat evaluations with a change in antibody status.

	**Patient**	**Clinical presentation**	**Panel 1 (result, level)**	**Panel 2 (result, level)**	**Time between evaluations (months)**	**Management changes**
Positive to negative, non-identical panels	Patient 1	Seizures and Encephalopathy	Encephalopathy Panel, serum (GAD-65: 0.05 nmol/L)	Epilepsy Panel, serum (negative)	5	None
	Patient 2	Seizures	Encephalopathy Panel, serum (VGKC: 0.22 nmol/L, LGi1: positive)	Epilepsy Panel, serum (negative)	11	Patient tapered off ASM following repeat negative evaluation
	Patient 3	Myopathy with anti-Ku antibodies	Encephalopathy Panel, serum (GAD-65: 0.19 nmol/L)	Paraneoplastic Panel, serum (negative)	3	None
	Patient 4	Diarrhea, weight loss, and behavior changes	Paraneoplastic Panel, serum (ARBi: 5.41 nmol/L)^*^	Paraneoplastic Panel, CSF (negative)	0.5	None
	Patient 5	Memory impairments	Encephalopathy Panel, serum (GAD-65: 0.08 nmol/L)	Encephalopathy Panel, CSF (negative)	3	None
	Patient 6	Encephalopathy with memory impairments	Epilepsy Panel, serum (ARBi: 0.05 nmol/L)	Encephalopathy Panel, serum and Paraneoplastic Panel, CSF (negative)	0.5	None
	Patient 7	Encephalopathy in setting of phosphaturic mesenchymal tumor	Paraneoplastic Panel, CSF (gAChR: 0.3 nmol/L)	Encephalopathy Panel, serum (negative)	0.5	None
Positive to negative, identical panels	Patient 8	Encephalitis in setting of parotid carcinoma	Encephalopathy Panel, serum (GAD-65: 0.08 nmol/L)	Encephalopathy Panel, serum (negative)	11	None
Negative to positive, non-identical panels	Patient 9	Autonomic dysfunction	Paraneoplastic Panel, serum (negative)	Encephalopathy Panel, serum (GAD-65: 0.20 nmol/L)	16	None
	Patient 10	Encephalopathy with psychiatric symptoms	Encephalopathy Panel, CSF (negative)	Encephalopathy Panel, serum (GAD-65: 0.07 nmol/L)	6	Plasmapheresis initiated following GAD-65 antibody level^**^

*ARBi, Acetylcholine receptor binding antibody; gAChR, Alpha 3-ganglionic acetylcholine receptor antibody; GAD-65, Glutamic acid decarboxylase 65 antibody; LGI1, Leucine-rich glioma-inactivated 1 antibody; VGKC, Voltage gated potassium channel antibody*.

* *Antibodies to dipeptidyl-peptidase-like protein-6 (DPPX) identified in this patient, but not reported on commercial test result*.

** *Immunotherapy change based off change in clinical symptoms*.

The transition in antibody status changed clinical management in two cases. In the first case, ASMs were stopped in a patient with leucine-rich glioma-inactivated 1 (LGI1) antibody associated seizures following panel transition from positive to negative. For the second case immunotherapy was started in an individual presenting with encephalopathy following a new positive glutamic acid decarboxylase 65 (GAD-65) antibody. There were no cases in which new antibody panel results correlated to a change in clinical diagnosis or prompted a malignancy screening.

## Discussion

Overlapping and repeat evaluations for suspected autoimmune and paraneoplastic disorders occurred frequently at our medical center, occurring in 15.9% of patients with testing in 2017. The results of these evaluations rarely differed from the initial evaluation and seldom influenced clinical management. Our study suggests that, at our center, there is wide variability in practice habits surrounding autoantibody evaluations, reflecting the increasingly complex nature of autoantibody testing.

Generally agreed upon practice standards include concurrent serum and CSF evaluations for suspected central nervous system autoimmune disorders—a practice that was only implemented a fraction of the time in this study. Even among the paired serum and CSF evaluations, panels were ordered in a “mix and match” fashion (e.g., epilepsy panels in the serum with encephalitis panels in the CSF). The vast majority of unmatched panels (84.3% of panels evaluated) were serum panels. There are numerous potential explanations for serum testing lacking a matched CSF sample. Lumbar punctures are invasive, time consuming procedures, which some patients may refuse in the outpatient setting. Additional medical factors, including body habitus, infection risk, and mental status, may limit the ability to perform a lumbar puncture. Lastly, each antibody panel requires a minimum volume of CSF for evaluation; often infectious and neoplastic studies are additionally needed from the same CSF sample. If the amount of CSF required for the intended studies is not calculated prior to the lumbar puncture, clinicians may have to choose between studies or risk a repeat procedure.

The practice of ordering overlapping evaluations—herein defined as multiple requests for antibody panels obtained <14 days apart, excluding paired serum and CSF evaluations—may reflect several factors. First, ordering clinicians are presented with a myriad of options when considering panel evaluations. This is compounded by frequent updates to panel contents and the interval development of panels for new indications. Anecdotal experience with clinicians suggests significant confusion regarding which panel is appropriate in a given scenario. All of these factors may lead clinicians to order multiple antibody panels in order to adequately evaluate patients with severe neurological impairment. Indeed, studies reviewing physician ordering practices and social consequences show a relative increase in false positive errors compared to false negative- implying clinicians regret the consequences of omitting testing that may lead to benefit more than the consequences of unnecessary testing (3). Comprehensive evaluation does not necessitate ordering multiple antibody panels, however; several established laboratories utilize immunohistochemistry as an initial screening tool which will detect known and unknown antibody binding. Patient 4 from Table 2 provides an example of this; at initial evaluation the patient was found to have an unidentified binding pattern on immunohistochemical staining. This was confirmed years later to be dipeptidyl-peptidase-like protein-6 (DPPX) antibodies, which was not commercially available at time of initial panel testing. Accidental reordering is an additional factor, as evidenced by 36% of overlapping evaluations consisting of identical panels in this study. Developing clinical decision support tools in electronic health records may be a viable strategy to mitigate many sources of overlapping evaluations.

The results of repeat evaluations rarely differed from those of initial testing. In this study, clinically insignificant GAD-65 antibodies (determined by low antibody levels and incompatible clinical phenotypes) represented the majority of antibody status conversions. GAD-65 antibodies are known to be present at low levels in healthy controls, systemic inflammatory or autoimmune disorders, and unrelated neurologic diseases (4, 5). When negating clinically insignificant GAD-65 antibodies, four repeat evaluations transitioned from positive to negative and zero transitioned from negative to positive. The latter indicates that initial negative autoantibody evaluations are sufficient for autoimmune and paraneoplastic antibody surveillance. Repeating identical testing follow initial negative evaluation is largely a flawed practice; management decisions should be influenced more heavily by clinical presentation with ancillary testing results than antibody status, as reflected in the 2016 consensus guidelines for diagnosing autoimmune encephalitis (6). Studies show providers appropriately initiate immunotherapy prior to panel results, however then discontinue immunotherapy following a negative antibody panel (7, 8). This practice is fraught with error as antibody negative autoimmune encephalitides are not uncommon (9).

Repeat testing following an initial positive antibody evaluation suggests a previous autoimmune or paraneoplastic disorder was known or suspected, therefore repeat testing would provide information to prompt changes to clinical management. There was a single case of seropositive conversion in which clinical management was altered, however the change in immunotherapy was based on clinical symptoms and not antibody status.

This pattern of widespread, redundant panel testing did not increase the sensitivity of detecting autoimmune or paraneoplastic diseases, nor did it alter clinical decision making. When juxtaposed against the expense of these panels, the value of this strategy in clinical practice is severely limited. Indeed, several retrospective studies have shown a significant proportion of autoantibody panels ordered are inappropriate based on clinical indication or other ancillary test results; this underscores the need for continued provider education in the field of autoimmune neurology and recognition of aggregate bias amongst ordering physicians (10). While many studies have shown low rates of antibody positivity with panel testing, increased clinical suspicion for primary autoimmune disorders improves panel sensitivity (11–13). Healthcare cost savings and autoantibody detection rates have additionally been shown to increase following the implementation of predictive likelihood scoring systems (14). Integrating decision support tools, such as the antibody prevalence in epilepsy and encephalopathy (APE2) score, into the electronic medical record and the resultant effect on physician ordering practices is an area needed in future study.

Based on the results of this study, the following recommendations for CNS autoimmune and paraneoplastic panel testing should be considered by clinicians.

1. Panel testing should be ordered as paired serum and CSF samples. This ensures the highest sensitivity for the greatest number of antibody mediated diseases—some of which are more sensitive in the CSF, others more sensitive in the serum.
2. Paired panel testing should be ordered as matching pairs. For example, encephalopathy panels in the serum will match antibodies most closely with encephalopathy panels in the CSF.
3. Overlapping panel evaluations are not advised if sending panel testing to a laboratory which performs tissue-based screening assays with reflexive testing; the screening assay will capture antibodies not specified in the panel and the appropriate reflexive testing will be performed.
4. If a clinician is considering ordering an overlapping evaluation and submitting samples to a laboratory without screening and reflexive assays, identify the antibody targets that differ between the panels. Revisit the clinical presentation of the patient and critically evaluate if the unique antibodies on either panel fit the clinical presentation of the patient.
5. Repeat autoantibody evaluations should be avoided, unless there is a significant change in the clinical status of the patient, or a specific antibody is highly suspected that was not evaluated for on the initial panel. This is especially cautioned against in individuals with prior negative autoimmune panel testing.
6. Repeat, identical antibody testing is not recommended for individuals with an appropriate initial evaluation with a negative result for the following indications: autoimmune encephalitis, epilepsy, movement disorders, and paraneoplastic disorders.
7. Repeat, identical antibody testing can be performed in seropositive individuals to assess for titer change or transition to seronegative status if there is a predetermined plan which influences clinical management, including but not limited to changes in ASMs, immunotherapies, or malignancy surveillance.
8. Isolated antibody testing should be reserved for the conditions outlined in recommendation seven; CNS autoimmune and paraneoplastic conditions should otherwise be evaluated with panel testing given the non-specific nature of presentation.

There are some limitations to our study. First, a single year of panel testing for individuals was reviewed and cross matched for repeat evaluations over a fixed time frame at our institution. It is possible that there was additional antibody testing prior to 2011 or that individuals may have had additional testing outside our hospital. Additionally, this retrospective observational study was performed on adult patients at a single tertiary level medical center; the results of this study and ensuing recommendations may not be generalizable to community hospitals, areas without neurologic expertise, or pediatric populations. Throughout the study time frame there were significant changes in commercially available panels, which may have altered ordering patterns. While this study did evaluate if new antibody testing results impacted clinical decision making, it did not address if repeat evaluations with identical results altered clinical decision making. Lastly, changes in clinical decision making were strictly defined for the purposes of this study and may not encompass all possible clinical decisions based on new antibody panel results.

## Conclusions

Ordering practices for suspected autoimmune encephalitis and paraneoplastic disorders are suboptimal with frequent overlapping antibody panel evaluations and non-paired serum/CSF samples at our center. Repeat autoantibody testing is a commonplace practice yet yielded novel information in only a minority of cases. These new results were, as a rule, clinically irrelevant and changed clinical decision making in <1% of cases. There is limited utility in these practice patterns. Future efforts need to be directed at the development and standardization of neurological autoimmune and paraneoplastic autoantibody testing practice standards.

## Data Availability Statement

The raw data supporting the conclusions of this article will be made available by the authors, without undue reservation.

## Ethics Statement

The studies involving human participants were reviewed and approved by UT Southwestern IRB. Written informed consent for participation was not required for this study in accordance with the national legislation and the institutional requirements.

## Author Contributions

SF participated in the conception of the study, research design, performance of the research, data analysis, and article writing. KB participated in the research design, performance of the research, and article modification. SV participated in the research design and article modification. All authors contributed to the article and approved the submitted version.

## Conflict of Interest

SF's fellowship is funded by the National Multiple Sclerosis Society. She has served as a consultant for EMD Serono. SV has served as a consultant for Alterity, Genentech, Catalyst and Sage Therapeutics. He has received research support from Dysautonomia International, BioHaven, Grifols and Quest Diagnostics (through a licensing contract). KB's fellowship was funded by the Siegel Rare Neuroimmune Association.

## Footnotes

^1^Panels requested include PAVAL, ENS1, ENC1, EPC1, EPS1, PAC1, ENCEC, EPIES, EPIEC, ENCES, DEMEC, ENS2, EPS2, DEMES. For a full description of panels, visit www.mayocliniclabs.com/test-catalog.
